# Diagnostic Challenges of Neurocysticercosis in a Non-endemic Country: A Case Report

**DOI:** 10.7759/cureus.103755

**Published:** 2026-02-17

**Authors:** Kota Yamaguchi, Yuki Sakaeyama, Taiki Tokuyama, Mitsuyoshi Abe, Nobuo Sugo

**Affiliations:** 1 Department of Neurosurgery, Faculty of Medicine, Toho University, Tokyo, JPN

**Keywords:** del brutto criteria, neurocysticercosis, non-endemic country, ring-enhancing lesion, seizure

## Abstract

Neurocysticercosis (NCC) is the most common parasitic infection of the central nervous system worldwide and a leading cause of acquired epilepsy in endemic regions. In contrast, it is considered rare in developed countries such as Japan, where it is classified as a non-endemic region. Owing to increasing global migration, NCC is being encountered more frequently in non-endemic settings, where atypical clinical and radiological presentations often lead to diagnostic difficulty, particularly when parasitic structures are not pathologically identified.

We report a case of NCC in a Nepalese immigrant in Japan with seizures and a solitary ring-enhancing brain lesion, diagnosed using established criteria in a non-endemic setting. A 31-year-old Nepalese man living in Japan presented with new-onset generalized tonic-clonic seizures. Neuroimaging revealed a solitary calcified intracerebral lesion in the right frontal lobe with ring enhancement and marked perilesional edema. No definite scolex was identified on imaging. Because the diagnosis remained uncertain, surgical resection was performed. Histopathological examination demonstrated an encapsulated cystic lesion with a three-layered architecture, but no definitive parasitic structures were identified. Microbiological stains were negative, and the Ki-67 labeling index was extremely low. Based on the Del Brutto diagnostic criteria, the patient fulfilled one major, two minor, and one epidemiological criterion, leading to a diagnosis of probable NCC. The postoperative course was uneventful, and no seizure recurrence or radiological relapse was observed during 12 years of follow-up.

This case illustrates that NCC can present as a solitary ring-enhancing intracranial lesion without identifiable parasitic structures in non-endemic countries. A comprehensive diagnostic approach integrating clinical history, neuroimaging findings, epidemiological background, pathological features, and established diagnostic criteria is essential for appropriate diagnosis in such settings.

## Introduction

Neurocysticercosis (NCC) is the most common parasitic infection of the central nervous system worldwide and a leading cause of acquired epilepsy in endemic regions [1,2]. It is caused by the larval stage of *Taenia solium* and remains a major public health problem in many low- and middle-income countries, particularly in Latin America, sub-Saharan Africa, and South and Southeast Asia [3,4]. Humans acquire NCC through fecal-oral transmission of *T. solium* eggs, typically via contaminated food or water. In contrast, NCC is considered rare in developed countries such as Japan, where it is classified as a non-endemic region [3].

However, increasing global migration has led to a growing number of NCC cases reported in non-endemic countries, posing diagnostic challenges for clinicians unfamiliar with the disease [3,4]. In such settings, NCC may present with atypical clinical and radiological features and is often misdiagnosed as a brain tumor, abscess, or other intracranial space-occupying lesion [5,6]. This diagnostic difficulty is further compounded when no identifiable parasite structures are observed pathologically [5].

Neuroimaging plays a central role in the diagnosis of NCC, yet its appearance varies widely depending on the number, location, and stage of the lesions, as well as the host inflammatory response [1,6]. Ring-enhancing lesions without visible cysts or scolex can closely mimic neoplastic or infectious conditions, particularly in non-endemic countries where NCC is not routinely considered in the differential diagnosis [2,5].

The diagnostic criteria proposed by Del Brutto provide a structured framework for the diagnosis of NCC by integrating clinical, epidemiological, radiological, immunological, and pathological findings [1]. These criteria are especially valuable in cases lacking direct parasitological confirmation [1,6].

Herein, we report a case of NCC in a Nepalese immigrant worker in Japan who presented with generalized seizures and a solitary ring-enhancing brain lesion without demonstrable parasite structures. Despite the absence of definitive pathological evidence of the parasite, a rational and comprehensive diagnostic approach based on clinical course, imaging findings, surgical observations, long-term follow-up, and established diagnostic criteria supported the diagnosis of NCC. This case highlights the importance of considering NCC in the differential diagnosis of intracranial lesions in non-endemic countries and underscores its educational value for neurosurgeons and clinicians practicing in increasingly multicultural societies.

## Case presentation

A 31-year-old man with no significant past medical history presented to the emergency department after a witnessed generalized tonic-clonic seizure lasting approximately one to two minutes. On the morning of admission, he was noted to have brief staring behavior followed by a headache prior to seizure onset. Emergency medical services were contacted immediately.

He had no prior history of epilepsy, immunodeficiency, or recent infection. The patient was born and raised in Nepal and had been living in Japan. On arrival, his consciousness was mildly impaired (Glasgow Coma Scale score: E4V3M5). Vital signs were as follows: blood pressure 170/100 mmHg, heart rate 112 beats/min, respiratory rate 24 breaths/min, and oxygen saturation 98% on room air. Electrocardiography showed a normal sinus rhythm. Neurological examination revealed no focal motor deficits, sensory disturbance, dysarthria, or meningeal signs. Pupils were equal (3 mm) and reactive to light bilaterally. Laboratory investigations demonstrated a C-reactive protein level of 0.4 mg/dL (reference range: <0.3 mg/dL) and a white blood cell count of 8,900/µL (3,300-8,600/µL), consistent with a mild inflammatory response. Hemoglobin was 18.3 g/dL (13.5-17.6 g/dL), and the platelet count was 329,000/µL (150,000-350,000/µL).

**Figure 1:**
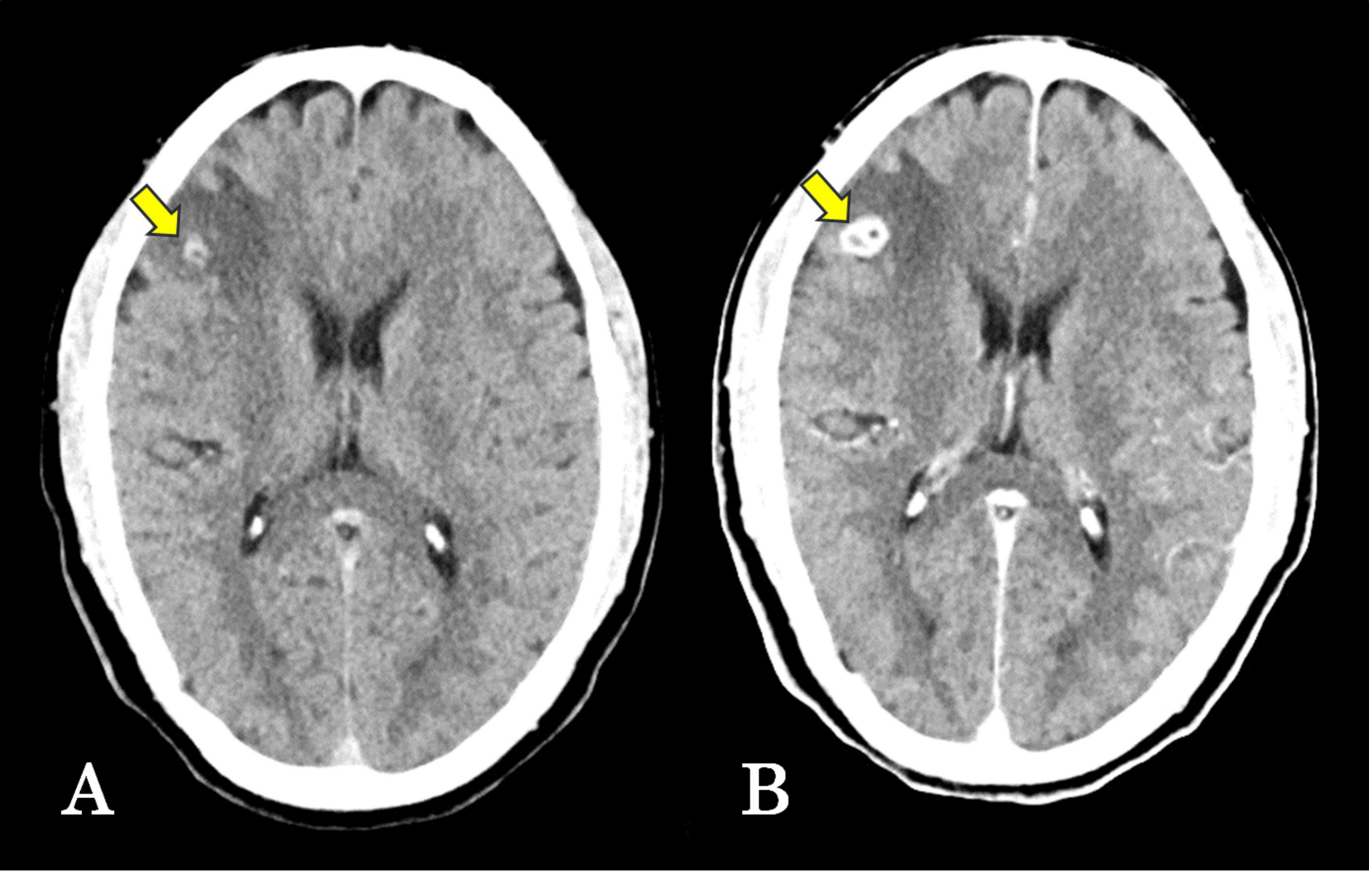
Computed tomography (CT) findings of the intracranial lesion (A) Non-contrast head CT showing a calcified mass lesion in the right frontal lobe. (B) Contrast-enhanced CT demonstrating ring enhancement of the lesion. The arrows indicate the lesion.

Non-contrast head computed tomography (CT), performed approximately two hours after symptom onset, revealed a calcified mass lesion in the right frontal lobe (Figure 1A). Contrast-enhanced CT demonstrated ring enhancement of the lesion (Figure 1B).

**Figure 2:**
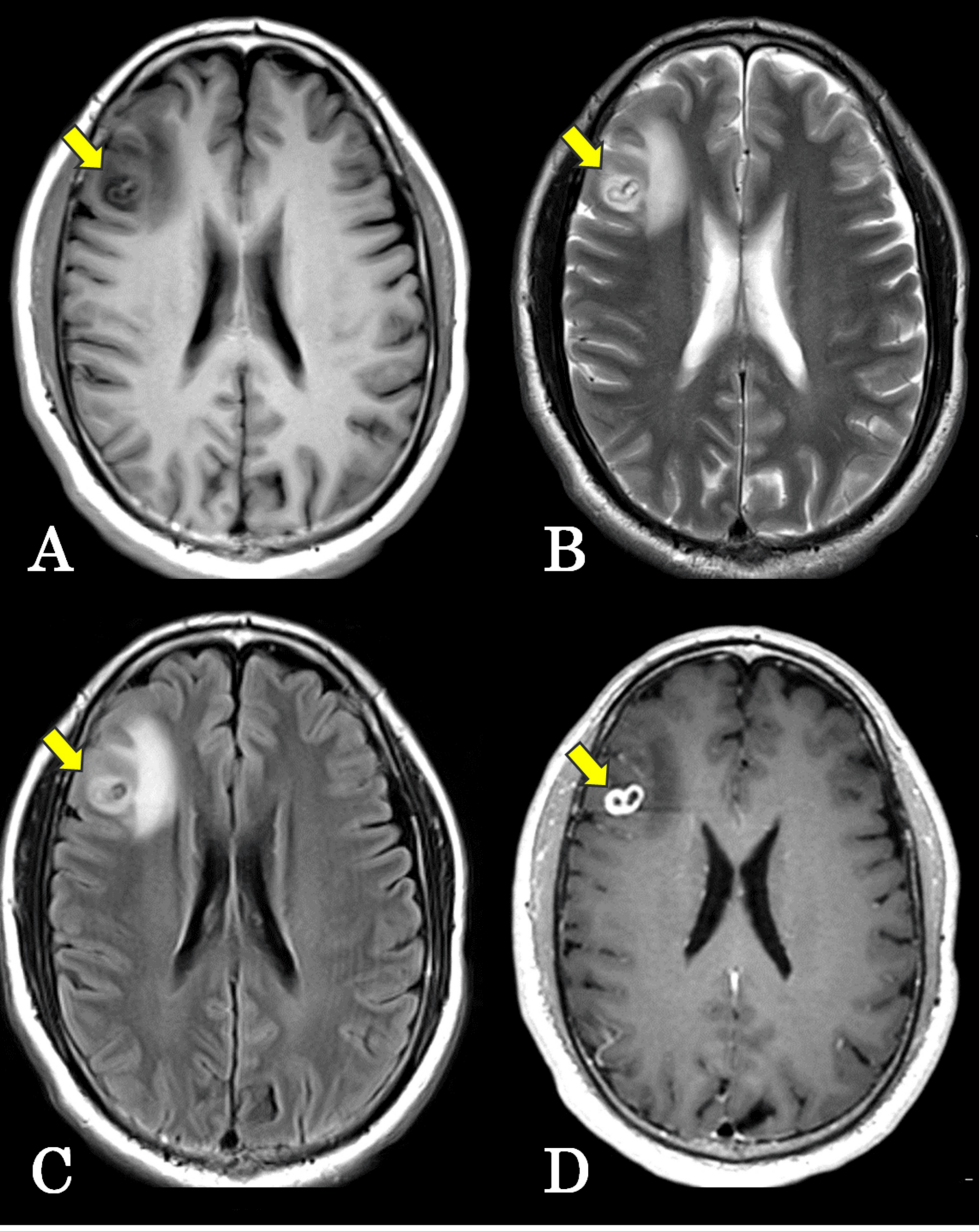
Magnetic resonance imaging (MRI) findings of the intracerebral lesion (A) T1-weighted MRI showing a hypointense lesion in the right frontal lobe. (B) T2-weighted MRI demonstrating marked perilesional edema. (C) Fluid-attenuated inversion recovery MRI showing prominent perilesional edema. (D) Gadolinium-enhanced T1-weighted MRI revealing a solitary cystic intracerebral lesion with ring-like enhancement. The arrows indicate the lesion.

On magnetic resonance imaging (MRI), the lesion was hypointense on T1-weighted images (Figure 2A). T2-weighted and fluid-attenuated inversion recovery images demonstrated marked perilesional edema extending into the surrounding white matter, resulting in mild local mass effect (Figures 2B, 2C). Gadolinium-enhanced T1-weighted images showed a solitary cystic intracerebral lesion measuring approximately 15 × 10 mm with ring-like enhancement (Figure 2D).

Although the imaging findings were non-specific, NCC was considered in the differential diagnosis, as this condition can present with imaging features similar to neoplastic or infectious intracranial lesions in non-endemic settings. No definite scolex was identified. Because the diagnosis remained uncertain, surgical intervention was performed two weeks after presentation. A right frontotemporal craniotomy was carried out. Intraoperatively, the lesion appeared as a firm, well-circumscribed intraparenchymal nodule. After complete excision, the specimen was incised extracorporeally, releasing a purulent-like fluid.

**Figure 3:**
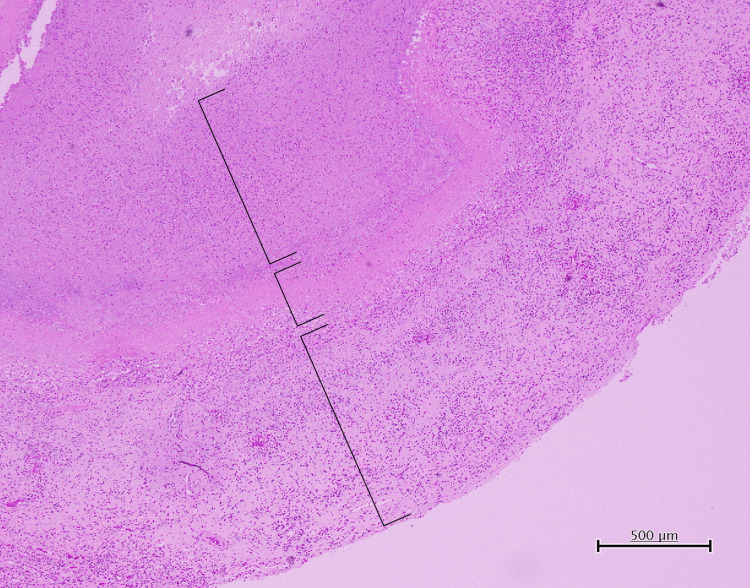
Histopathological findings of the intracerebral lesion Histopathological examination revealed an encapsulated cystic lesion with a distinct three-layered structure, consisting of an inner necrotic cavity without parasite-specific structures, a fibrous inflammatory capsule, and outer brain parenchyma with mild reactive gliosis. The lines indicate the three respective layers.

Histopathological examination demonstrated an encapsulated cystic intracerebral lesion with a distinct three-layered architecture (Figure 3).

The inner layer consisted of a cystic cavity containing necrotic debris. No parasite-specific structures were identified, and therefore definitive attribution to a specific etiology, including parasitic infection, was not possible. The middle layer comprised a fibrous capsule reflecting a host response, accompanied by capillary proliferation and inflammatory cell infiltration predominantly composed of lymphocytes and plasma cells. The outer layer consisted of adjacent brain parenchyma with mild reactive gliosis. No microorganisms were detected on periodic acid-Schiff, Grocott methenamine silver, or Ziehl-Neelsen staining. The surrounding brain tissue showed mild glial proliferation without significant nuclear atypia or mitotic activity, and the Ki-67 labeling index was extremely low. Overall, these histological features were compatible with a three-layered structure suggestive of NCC.

However, no definitive parasitic elements, including a scolex, were identified, and therefore, a pathological confirmation of cysticercosis could not be established. Based on the diagnostic criteria proposed by Del Brutto [1], this case fulfilled one major criterion (a cystic lesion with calcification on neuroimaging), two minor criteria (seizure onset and neuroimaging findings compatible but non-specific for NCC), and one epidemiological criterion (residence in an endemic area). Accordingly, the diagnosis of probable NCC was made (Table 1) [1].

**Table 1:** Application of Del Brutto criteria to the present case with stage correlation Application of the Del Brutto diagnostic criteria showing fulfillment of one major, two minor, and one epidemiological criterion, leading to a diagnosis of probable NCC despite the absence of definitive parasitic elements. NCC: neurocysticercosis; CT: computed tomography; MRI: magnetic resonance imaging; CSF: cerebrospinal fluid

Del Brutto criteria		Findings in the present case	Fulfilled	Correlation with disease stage
I. Absolute criteria	Imaging demonstration of scolex	Scolex not identified on CT or MRI	No	Scolex is typically preserved only in the vesicular (viable) stage
	Histopathological demonstration of cysticercus	No definitive cysticercus or scolex identified; cyst wall–like structure only	No	Parasite structures are often absent in late degenerative stages
	Direct visualization of intraocular cysticercosis	Not evaluated	No	Not applicable
II. Major criteria	Typical neuroimaging findings of NCC	Cystic lesion with intracranial calcification	Yes	Characteristic of the calcified stage
	Positive serum or CSF anti-*Taenia solium* antibodies	Not examined	Not assessed	Not applicable
	Resolution of lesions after antiparasitic therapy	Not treated	Not assessed	Not applicable
	Typical intracranial calcifications suggestive of NCC	Solitary calcified lesion	Yes	Consistent with the calcified stage
III. Minor criteria	Clinical manifestations suggestive of NCC	New-onset epilepsy	Yes	Common from the colloidal vesicular stage onward
	Neuroimaging findings are compatible but not specific for NCC	Ring-enhancing cystic lesion	Yes	Typical of late-stage NCC
	CSF abnormalities	Not evaluated	Not assessed	Not applicable
IV. Epidemiologic criteria	Residence in or exposure to an endemic area	Born and raised in Nepal	Yes	Applicable to all disease stages
Diagnostic category		One major + two minor + one epidemiologic criterion	Probable NCC	Late degenerative (granular–nodular to calcified) stage

The postoperative course was uneventful. The patient experienced no further seizures, and during a 12-year follow-up period, there was no radiological evidence of lesion recurrence.

## Discussion

NCC is the most common parasitic infection of the central nervous system worldwide; however, it remains rare in non-endemic countries such as Japan [1,4]. Consequently, NCC is often overlooked in the differential diagnosis of intracranial space-occupying lesions, particularly when radiological findings are atypical and parasitic structures are not directly identified. This lack of familiarity frequently results in diagnostic delay or misdiagnosis in non-endemic settings. In the present case, the patient presented with new-onset generalized seizures and a solitary intracerebral lesion with ring enhancement and calcification. These imaging features are non-specific and substantially overlap with those of brain abscesses, metastatic tumors, and high-grade gliomas, which are usually prioritized in the diagnostic process in non-endemic countries [5,6]. As a result, surgical intervention was selected before a definitive diagnosis could be established.

Similar diagnostic challenges have been reported in non-endemic countries, including Japan, where NCC has been incidentally detected in patients with suspected metastatic brain tumors based on epidemiological exposure and characteristic imaging findings, even when serological tests were negative. These reports underscore the importance of integrating travel history and radiological patterns to avoid misinterpretation of cystic or ring-enhancing lesions as neoplastic disease [7].

To address such diagnostic challenges, Del Brutto proposed standardized diagnostic criteria that integrate clinical manifestations, neuroimaging findings, epidemiological exposure, immunological tests, and pathological evidence [1]. Applying these criteria to the present case, the absolute criteria were not fulfilled because definitive parasitic structures, such as a scolex, were not identified pathologically. Nevertheless, the case satisfied one major criterion (a cystic lesion with calcification on neuroimaging), two minor criteria (seizure onset and imaging findings compatible but non-specific for NCC), and one epidemiological criterion (residence in an endemic area). Therefore, according to the Del Brutto criteria, the diagnosis of probable NCC was considered appropriate.

A critical aspect of this case is the interpretation of pathological findings in the context of disease stage. NCC is known to evolve through four pathological stages: vesicular, colloidal vesicular, granular-nodular, and calcified [1,5]. In the vesicular (viable) stage, the cysticercus structure and scolex are preserved, the inflammatory reaction is minimal, and the characteristic “hole-with-dot” sign may be observed on neuroimaging. During the colloidal vesicular stage, the parasite degenerates, cyst fluid becomes turbid, and a marked inflammatory response develops, often accompanied by granuloma formation and prominent clinical symptoms such as seizures [5,6]. As the disease progresses to the granular-nodular stage, parasitic structures are largely destroyed and replaced by fibrotic and granulomatous tissue, while inflammation gradually subsides. Finally, in the calcified stage, the parasite is completely necrotic and replaced by a calcified nodule, with little or no residual inflammation. Despite being inactive, calcified lesions may persist as epileptogenic foci [1,4].

In the present case, histopathological examination revealed a cyst wall-like structure with a three-layered architecture but no identifiable scolex or viable parasitic elements, and neuroimaging demonstrated calcification. These findings are incompatible with the vesicular stage and argue against active infection. Instead, they are most consistent with a late degenerative phase, corresponding to the granular-nodular to calcified stage, in which the parasite has already been destroyed by host immune responses. From this perspective, the absence of a scolex should not be regarded as a diagnostic weakness but rather as a reflection of the natural history of NCC in its late stages.

The long-term clinical course further supports this interpretation. Following surgical removal of the lesion, the patient remained seizure-free with no radiological recurrence over a 12-year follow-up period, without the need for antiparasitic therapy. Such a benign and stable course is inconsistent with malignant neoplasms or progressive inflammatory diseases and is compatible with inactive or end-stage NCC lesions [4].

This case highlights several important clinical lessons. First, NCC should be considered in the differential diagnosis of solitary ring-enhancing intracranial lesions even in non-endemic countries, particularly in patients with epidemiological risk factors. Second, the absence of a scolex or definitive parasitic structures should not preclude the diagnosis when clinical, radiological, and epidemiological features are supportive, and the disease stage is appropriately considered. Finally, the Del Brutto diagnostic criteria, when applied in conjunction with an understanding of stage-dependent pathology, provide a rational and practical framework for diagnosing NCC in challenging cases.

With increasing global migration, clinicians in non-endemic regions such as Japan are likely to encounter NCC more frequently. Awareness of its stage-dependent presentations and systematic application of established diagnostic criteria are essential to avoid misdiagnosis and unnecessary invasive interventions.

## Conclusions

NCC should be considered in the differential diagnosis of intracranial ring-enhancing lesions, even in non-endemic countries such as Japan, particularly in patients with epidemiological risk factors. This case demonstrates that the absence of a scolex or definitive parasitic structures does not exclude the diagnosis, especially in late degenerative stages of the disease. Application of the Del Brutto diagnostic criteria, together with an understanding of stage-dependent pathology, enables a rational and clinically meaningful diagnosis. Increased awareness of NCC is essential to avoid misdiagnosis and unnecessary interventions in increasingly multicultural clinical settings.

## References

[1] Del Brutto OH (2014). Neurocysticercosis. Neurohospitalist.

[2] DeGiorgio CM, Medina MT, Durón R, Zee C, Escueta SP (2004). Neurocysticercosis. Epilepsy Curr.

[3] Mewara A, Goyal K, Sehgal R (2013). Neurocysticercosis: a disease of neglect. Trop Parasitol.

[4] Del Brutto OH (2022). Human neurocysticercosis: an overview. Pathogens.

[5] Pittella JE (1997). Neurocysticercosis. Brain Pathol.

[6] Nash TE, Garcia HH (2011). Diagnosis and treatment of neurocysticercosis. Nat Rev Neurol.

[7] Kinouchi T, Morishima Y, Uyama S, Miyamoto T, Horiguchi H, Fujimoto N, Ueta H (2021). Neurocysticercosis in a Japanese woman with lung cancer who repeatedly visited endemic countries. BMC Infect Dis.

